# The mechanism of lncRNA PVT1 in oral squamous cell carcinoma

**DOI:** 10.3389/fonc.2026.1754726

**Published:** 2026-04-28

**Authors:** Qunli Ren, Xiaolan Li, Yuxiang Li, Xiangye Hu, Kangyi Liu, Sheng Tang, Zihan Zhang, Zhihui Wen, Miao Wang, Qian Wang, Jianguo Liu, Bin Chen

**Affiliations:** 1 School of Stomatology, Zunyi Medical University, Zunyi, Guizhou, China; 2 Key Laboratory of Microbial Resources and Drug Development of the Education Department of Guizhou Province, Zunyi Medical University, Zunyi, Guizhou, China; 3 Zunyi Laboratory of Oral Diseases Research, Zunyi Medical University, Zunyi, Guizhou, China

**Keywords:** clinical significance, diagnostic biomarker, lncRNA PVT1, molecular mechanism, oral squamous cell carcinoma

## Abstract

Oral squamous cell carcinoma, a common malignancy of the head and neck, is characterized by high rates of recurrence, metastasis, and chemoresistance, contributing to its persistently low five-year survival rate. Long non-coding RNA Plasmacytoma Variant Translocation 1(lncRNA PVT1), a significant oncogenic non-coding RNA, is frequently overexpressed in various human malignancies. Accumulating evidence highlights the central contribution of lncRNA PVT1 to OSCC pathogenesis through its regulation of essential oncogenic phenotypes, such as cell proliferation, invasion, metastatic dissemination, immune evasion, and chemoresistance. This review systematically summarizes the expression profile, functional mechanisms, and clinical significance of lncRNA PVT1 in OSCC, with a specific focus on molecular pathways such as the competitive endogenous RNA mechanism, induction of epithelial-mesenchymal transition, modulation of key signaling pathways, and upstream regulation by m6A methylation. Furthermore, the potential of lncRNA PVT1 as a novel diagnostic biomarker and therapeutic target is discussed.

## 1 Introduction​

Oral Squamous Cell Carcinoma (OSCC) is the most prevalent subtype of head and neck malignancies, accounting for over 90% of all oral cancer cases. According to GLOBOCAN 2022 statistics, there were approximately 389,485 new OSCC cases and 188,230 deaths reported globally, with the incidence rate demonstrating a consistent upward trend (1, 2). However, despite continuous advancements in diagnostic and therapeutic techniques such as surgery, radiotherapy, chemotherapy, and immunotherapy, the five-year survival rate for OSCC remains unsatisfactory, which is largely fueled by local recurrence, lymph node metastasis, and chemoresistance (3–5). While natural active compounds have recently emerged as promising candidates for oral cancer therapy (6), harnessing this potential however requires the identification of specific biomarkers and effective therapeutic targets to improve clinical outcomes. Long non-coding RNAs (lncRNAs), which exceed 200 nucleotides in length and lack protein-coding capacity, play pivotal roles in regulating gene expression epigenetically, transcriptionally, and post-transcriptionally (7). Their dysregulation in cancer can disrupt these processes, thereby acting as drivers of tumorigenesis and progression (8). LncRNA PVT1, an oncogene located near the c-Myc gene on chromosome 8 (9, 10), is aberrantly overexpressed in numerous cancers, including gastric, lung, hepatic, and breast carcinomas (11–16). Its upregulation is closely associated with advanced tumor node metastasis (TNM) stage, higher pathological grade, lymph node metastasis, and poor survival. Moreover, elevated lncRNA PVT1 expression strongly predicts reduced overall survival and progression-free survival in OSCC patients (17–20). By synthesizing the role of lncRNA PVT1 in OSCC across molecular mechanisms and clinical translation, this review consolidates current knowledge and informs future strategies for precise management.

## 2 Mechanisms of lncRNA PVT1 in OSCC

**Figure 1:**
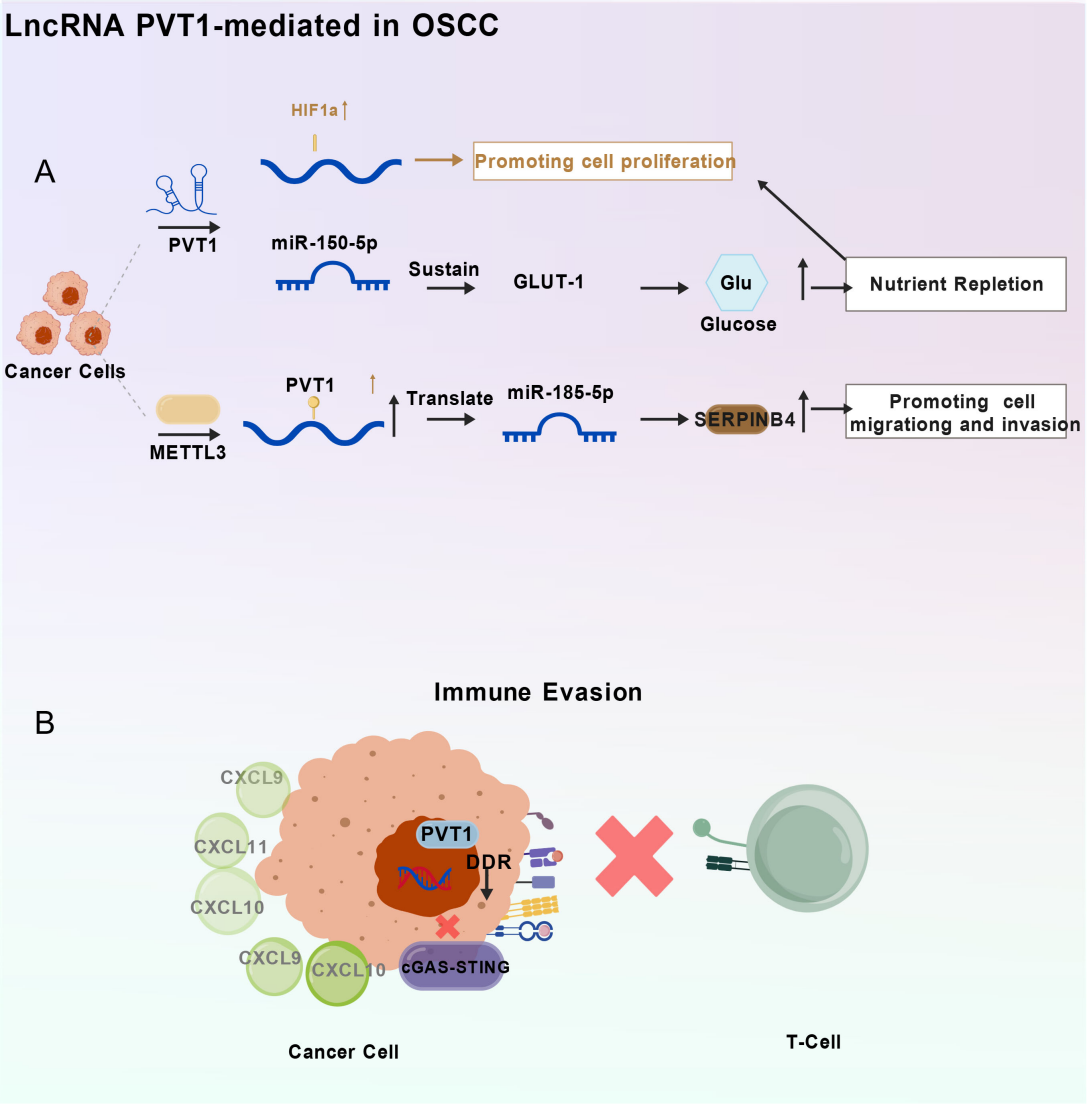
The molecular mechanisms of LncRNA PVT1 in promoting OSCC progression. **(A)** PVT1 drives tumor cell proliferation and metastasis through multiple pathways. On one hand, PVT1 sustains the expression of GLUT-1, a key glucose transporter, potentially through sponging miR-150-5p or interacting with the HIF1α signaling pathway, thereby enhancing glucose uptake and nutrient repletion for cancer cell growth. On the other hand, PVT1, whose stability is enhanced by METTL3-mediated m6A modification, sponges miR-185-5p, leading to the derepression of SERPINB4, which promotes cancer cell migration and invasion. **(B)** PVT1 facilitates immune evasion by suppressing the tumor immune microenvironment. High levels of PVT1 inhibit the cGAS-STING pathway, which is crucial for triggering innate immune responses. This inhibition leads to the downregulation of key T-cell chemoattractants, such as CXCL9, CXCL10, and CXCL11. Consequently, the recruitment of anti-tumor T-cells to the tumor site is impaired, allowing cancer cells to evade immune surveillance. DDR, DNA Damage Response. GLUT-1, Glucose Transporter 1. SERPINB4, Serpin Family B Member 4. cGAS-STING, cyclic GMP-AMP Synthase-Stimulator of Interferon Genes. OSCC, Oral Squamous Cell Carcinoma.

LncRNA PVT1 does not exert its oncogenic effects in OSCC through a single pathway, but rather by regulating a complex molecular network that governs the malignant biological behaviors of tumor cells (**Figure 1**). Its core mechanisms primarily include functioning as a competitive endogenous RNA (ceRNA) to sequester microRNAs, modulating key signaling pathways, regulating upstream by N6-methyladenosine (m6A) RNA methylation, remodeling the tumor microenvironment (TME), inducing epithelial-mesenchymal transition (EMT), and maintaining cancer stem cell (CSC) stemness.

### 2.1 Promoting tumor cell proliferation and inhibiting apoptosis

Tumor cell proliferation and apoptosis are central to cancer development (21), lncRNA PVT1 disrupts the balance between proliferation and apoptosis in OSCC cells via various mechanisms, thereby providing favorable conditions for tumor growth.

#### 2.1.1 The molecular sponging mechanism of ceRNA

LncRNA PVT1 functions primarily as a competing endogenous RNA (ceRNA) in OSCC, acting as a molecular sponge for tumor-suppressive microRNAs (miRNAs). By sequestering these miRNAs, PVT1 relieves their repression of downstream oncogenic targets, thereby broadly promoting tumor cell proliferation and survival (17–23). This core ceRNA function is executed through several well-defined regulatory axes. In the PVT1/miR-7-5p/CDKL1 axis, lncRNA PVT1 sponges miR-7-5p, leading to derepression of the cell cycle regulator cyclin-dependent kinase-like 1 (CDKL1), which accelerates the G1/S transition and enhances OSCC cell proliferation in CAL-27 and Tca-83 cell lines (24). Additionally, in the PVT1/miR-185-5p/SERPINB4 axis, lncRNA PVT1 binds miR-185-5p, leading to Serpin Family B Member 4 (SERPINB4) upregulation, a driver of proliferation, migration, and invasion in OSCC cells (HEK293T, CAL-27, and Tca-83), an effect further augmented by methyltransferase-like 3 (METTL3)-mediated m6A modification, which stabilizes PVT1 and enhances its miRNA-sequestering efficiency (18). The specific mechanism of this axis in facilitating extracellular matrix degradation and metastasis is elaborated in Section 2.2.2. In the PVT1/miR-150-5p/GLUT-1 axis, PVT1 sequesters miR-150-5p to upregulate glucose transporter 1 (GLUT-1), enhancing glycolysis and fueling OSCC cell (SCC-25 and CAL-27) proliferation and survival (22). This axis’s pivotal role in promoting cytoskeletal rearrangement and *in vivo* metastasis is further explored in Section 2.2.2. Moreover, in PVT1/miR-194-5p/HIF1α axis, lncRNA PVT1 sponges miR-194-5p to upregulate hypoxia-inducible ​factor 1-α (HIF1α), supporting proliferation and chemoresistance under hypoxic conditions in SCC9, CAL-27, and HEK-293T cells (17). In summary, lncRNA PVT1 acts as a master regulatory hub within a ceRNA network, coordinating multiple miRNA-target axes to orchestrate fundamental oncogenic processes in OSCC.

#### 2.1.2 Modulation of signaling pathways​​

Beyond its ceRNA function, lncRNA PVT1 modulates key oncogenic signaling pathways to drive OSCC progression. One primary mechanism involves activation of the Wnt/β-catenin pathway. This highly conserved pathway, which orchestrates cell-cell communication during normal proliferation and embryonic development, is frequently dysregulated through mutation or aberrant activation in cancer. Its dysfunction is closely linked to the pathogenesis of a wide range of malignancies (25, 26). Studies conducted in head and neck squamous cell carcinoma (HNSCC) cell lines (Tu686 and FaDu) demonstrated that lncRNA PVT1 overexpression suppresses phosphorylated GSK3β (p-GSK3β), which stabilizes β-catenin, facilitates its nuclear translocation, and subsequently activates downstream proliferative gene transcription. The pro−proliferative effect of PVT1 was reversed by the Wnt/β−catenin inhibitor iCRT14, accompanied by restoration of the epithelial marker E−cadherin and downregulation of the mesenchymal marker vimentin, indicating that lncRNA PVT1 promotes both cell proliferation and EMT through Wnt/β−catenin signaling (19). Concurrently, lncRNA PVT1 amplifies the transforming growth factor−beta (TGF−β) pathway. In OSCC cells (e.g., CAL−27), lncRNA PVT1 upregulates both the mRNA and secreted protein levels of TGF−β, whereas its knockdown significantly attenuates TGF−β expression and secretion. This observation suggests that lncRNA PVT1 drives OSCC progression by enhancing TGF−β signaling, a context-dependent​ pathway that suppresses tumors in early stages but promotes tumor growth and immunosuppression in advanced disease (27, 28).

#### 2.1.3 Upstream regulation by m6A RNA methylation

The aberrantly high expression of lncRNA PVT1 is not incidental but is precisely regulated by the dynamic and reversible RNA modification m6A, forming a bidirectional cross-regulatory network that critically enhances the complexity and context-specificity of its oncogenic functions. In OSCC, the methyltransferase METTL3 installs m6A marks on PVT1 transcripts, which are recognized by reader proteins such as YTHDF1/2 (29), thereby enhancing PVT1 RNA stability and driving downstream oncogenic pathways, including the PVT1/miR-185-5p/SERPINB4 axis (18). A similar METTL3-mediated stabilizing mechanism promotes PVT1 expression and tumor progression in prostate cancer (30). Intriguingly, the demethylase ALKBH5 can also upregulate PVT1 in certain cancers, such as osteosarcoma, lung, and ovarian cancer, by removing m6A modifications, highlighting the context-dependent nature of this regulatory layer (31–33). Beyond being a substrate, PVT1 can actively influence the epitranscriptome by recruiting the m6A reader YTHDC1 to specific target mRNAs, such as IL-33 in bronchopulmonary dysplasia, thereby enhancing their m6A modification and stability and amplifying pathological signaling in a positive feedback loop (34). This regulatory axis is also crucial for maintaining stemness: in epidermal stem cells, m6A-modified PVT1 stabilizes MYC protein to sustain self-renewal (35), while glioma stem cells exhibit a distinct m6A modification pattern on PVT1, underscoring its contribution to stem cell state maintenance (36). Collectively, the intricate crosstalk between m6A methylation and PVT1 represents a sophisticated, bidirectional regulatory circuit that fine-tunes gene expression in development and disease, offering a promising target for therapeutic intervention.

### 2.2 Promoting tumor cell invasion and metastasis

The invasion and metastasis of tumor cells represent a major cause of poor prognosis in OSCC patients. LncRNA PVT1 enhances the invasive and metastatic capabilities of OSCC cells through various mechanisms, including the induction of EMT and the competing ceRNA mechanism, ultimately driving disease progression.

#### 2.2.1 Induction of EMT​

EMT is central to conferring migratory and invasive capabilities upon tumor cells. This process is hallmarked by the loss of typical junctions and apicobasal polarity, along with the gain of front-rear polarity, in epithelial cells undergoing the transition. Molecularly, it involves the downregulation of epithelial markers (like E-cadherin and ZO-1) and the upregulation of mesenchymal markers (like N-cadherin and Vimentin) (37). LncRNA PVT1 promotes invasion and metastasis in OSCC by inducing EMT through various mechanisms. For instance, its knockdown in HSC2 and SAS cells suppressed migration and invasion by reversing the EMT marker profile (upregulating E-cadherin and ZO-1 while downregulating N-cadherin and Vimentin) (38). Conversely, lncRNA PVT1 overexpression produced the opposite effects. Additionally, in FaDu and Tu686 cells, lncRNA PVT1 overexpression was shown to activate the Wnt/β-catenin pathway to drive EMT, an effect reversible upon pathway inhibition (19).

#### 2.2.2 Sponge absorption capacity

Building upon the established ceRNA network of PVT1 that drives core oncogenic phenotypes (see Section 2.1.1), its function as a molecular sponge is particularly critical in orchestrating the invasive and metastatic cascade of OSCC. LncRNA PVT1 achieves this by fine-tuning specific axes within its ceRNA network to modulate pro-invasive cellular processes (**Figure 1A**). Primarily, the PVT1/miR-150-5p/GLUT-1 axis​ promotes metastasis not only by fueling glycolysis but also by inducing cytoskeletal rearrangement, thereby directly enhancing cell migration and invasion in OSCC cell lines such as SCC-090 and CAL-27. This *in vivo* significance is corroborated by the suppression of pulmonary metastatic nodules upon PVT1 silencing, which coincides with reduced GLUT1 expression in lung tissues (22). Concurrently, the PVT1/miR-185-5p/SERPINB4 axis​ drives invasion by facilitating extracellular matrix (ECM) degradation, SERPINB4 indirectly potentiates matrix metalloproteinase (MMP) activity, and PVT1 knockdown attenuates this invasive capability—a phenotype rescued by SERPINB4 overexpression or miR-185-5p inhibition (18). Furthermore, the PVT1/miR-7-5p/CDKL1 axis​ contributes to the metastatic phenotype by derepressing CDKL1, which enhances the migratory and invasive abilities of OSCC cells like CAL-27 and Tca-83, establishing CDKL1 as a key downstream effector in PVT1-mediated metastasis (24). Collectively, these findings delineate how PVT1, via its ceRNA function, coordinately remodels cellular metabolism, degrades the ECM, and enhances motility to drive OSCC dissemination.

### 2.3 ​Modulation of the tumor microenvironment

The TME, constituted by cancer-associated fibroblasts (CAFs), immune cells, the ECM, and cytokines (39), constitutes a fundamental determinant of tumorigenesis and cancer progression. Dysregulation of the TME is intricately involved in key oncogenic processes, such as tumor cell proliferation, evasion of apoptosis, metastasis, and drug resistance (40, 41). LncRNA PVT1 is recognized for modulating key components of the TME, thereby fostering a permissive environment for the growth and metastasis of OSCC cells.

#### 2.3.1 Promoting the proliferation of CAFs

CAFs, the predominant stromal cells in the TME, promote tumor growth and metastasis through the secretion of cytokines such as TGF-β and IL-6. Supporting this, Meng et al. (2024) (28) reported that lncRNAPVT1 knockdown in a nude mouse xenograft model of OSCC significantly reduced CAF density (as indicated by α-SMA positive staining) and slowed tumor growth. Mechanistically, the study demonstrated that lncRNA PVT1 upregulates the mRNA and protein expression of TGF-β and IL-6 in OSCC cells. Consequently, lncRNA PVT1 fosters a TME conducive to CAF proliferation and ultimately driving OSCC progression (28).

#### 2.3.2 ​Inhibiting immune cell infiltration

Immune escape is a critical mechanism by which tumor cells evade immune surveillance. The dynamic crosstalk between cancer cells and immune cells shapes a pro-tumorigenic microenvironment that enables evasion of immune surveillance and is permissive for immune tolerance (42). LncRNA PVT1 facilitates immune escape in OSCC by suppressing T-cell infiltration (**Figure 1B**). Qin et al. (2023) (43) reported that silencing lncRNA PVT1 significantly enhanced the intratumoral CD8+ T-cell infiltration and inhibited tumor growth, which they attributed to an impaired ability to recruit these immune cells. Mechanistically, lncRNA PVT1 suppresses the DNA damage response (DDR), which consequently attenuates the cGAS-STING–chemokine signaling axis. This leads to reduced expression and secretion of T-cell chemoattractants such as CXCL9, CXCL10, and CXCL11, ultimately preventing the effective recruitment of CD8+ T cells into the tumor bed (43).

### 2.4 Induction of chemoresistance

**Figure 2:**
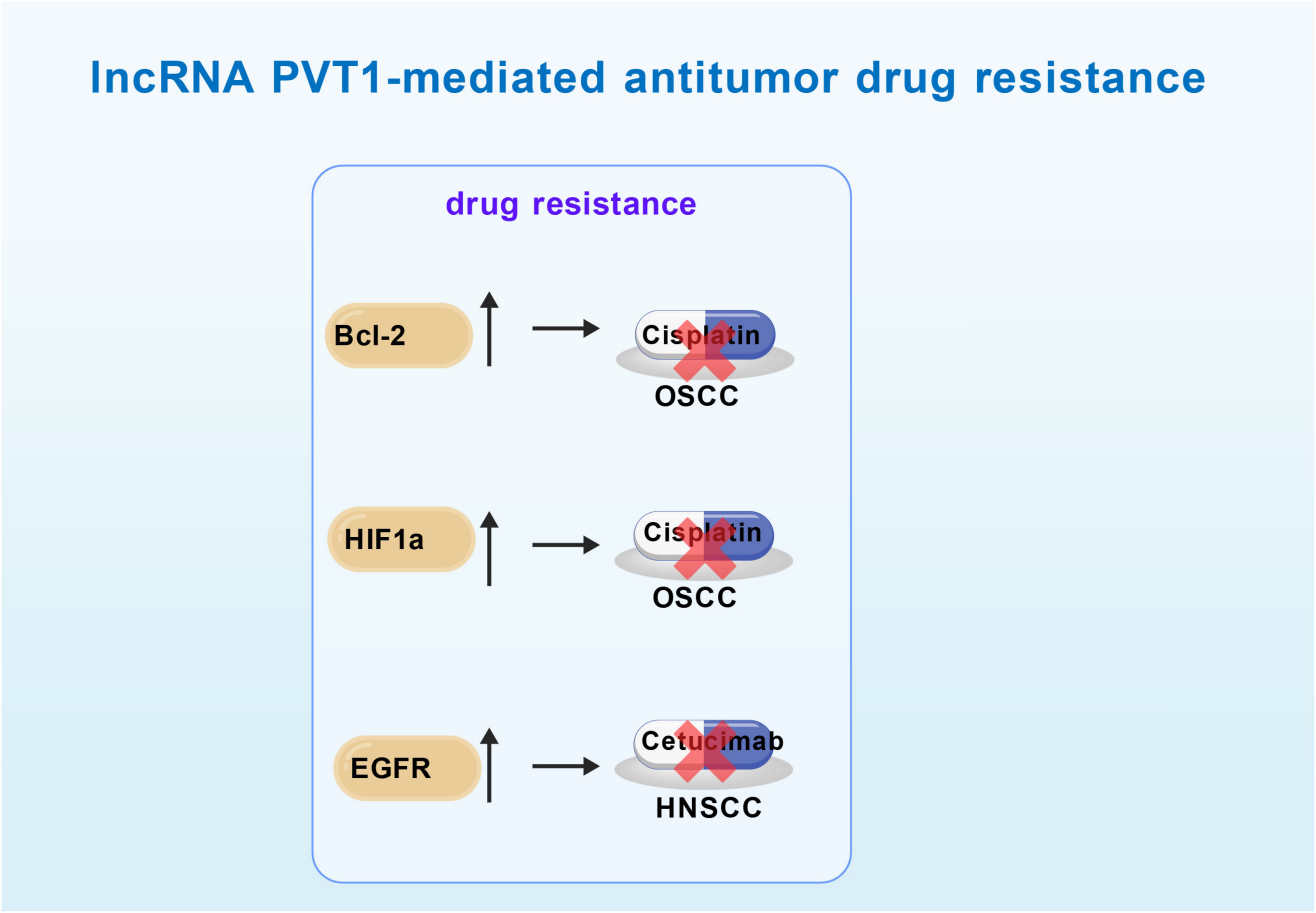
The mechanisms of LncRNA PVT1-mediated antitumor drug resistance in OSCC. LncRNA PVT1 promotes resistance to Cisplatin and Cetuximab in OSCC by upregulating the anti-apoptotic protein Bcl-2、EGFR and HIF1α. OSCC, Oral Squamous Cell Carcinoma. Bcl-2, B-cell lymphoma 2. HIF1α, Hypoxia-inducible factor 1-alpha. EGFR, Epidermal Growth Factor Receptor.

Chemotherapy remains a cornerstone in the clinical management of OSCC. However, the development of chemoresistance poses a major challenge to its efficacy. The lncRNA PVT1 has been strongly implicated in chemoresistance of OSCC cells, particularly to Cisplatin and Cetuximab (**Figure 2**).

#### 2.4.1 Cisplatin resistance

Cisplatin is a first-line chemotherapeutic agent for OSCC, but its efficacy is often limited by the development of resistance, primarily through mechanisms such as enhanced DNA damage repair, increased anti-apoptotic capacity, and elevated drug efflux. Wang et al. (2020) (17) demonstrated that lncRNA PVT1 contributes to cisplatin resistance through dual mechanisms. Their study showed that lncRNA PVT1 acts as a molecular sponge for miR-194-5p, leading to the upregulation of HIF1α. This enhances the transcription of DNA repair proteins and facilitates the repair of cisplatin-induced DNA damage in OSCC cells. Simultaneously, lncRNA PVT1 activates the PI3K/AKT signaling pathway, upregulates the anti-apoptotic protein Bcl-2, and consequently inhibits cisplatin-induced apoptosis. Together, these mechanisms underscore the role of PVT1 in promoting chemoresistance.

#### 2.4.2 Cetuximab resistance

Cetuximab plus platinum-based chemotherapy is employed as a systemic treatment regimen in the setting of recurrent or metastatic OSCC (44). Unfortunately, the acquisition of resistance compromises its clinical efficacy.​ Yang et al. (2021a) (45) demonstrated that elevated expression of lncRNA PVT1 is closely associated with cetuximab resistance in HNSCC cell lines SCC-9, SCC4, and CAL27. LncRNA PVT1 functions as a ceRNA by sequestering miR-124-3p, thereby upregulating the expression of its target gene EGFR. Increased EGFR expression enhances cellular resistance to cetuximab. Silencing PVT1 or overexpressing miR-124-3p significantly restored cetuximab sensitivity in HNSCC cells (45).

### 2.5 Maintaining OSCC cancer stem cell stemness

Cancer stem cells (CSCs), a small subset of cells within tumors, possess self-renewal and multi-lineage differentiation capabilities, and are closely associated with tumor recurrence, metastasis, and chemoresistance (46). LncRNA PVT1 has been identified as a master upstream regulator orchestrating the stemness of OSCC- initiating CSCs, with its dysregulation being tightly linked to the malignant progression of OSCC. LncRNA PVT1 can maintain OSCC CSC stemness by regulating relevant signaling pathways. Qin et al. (2023) (43) demonstrated that lncRNA PVT1 expression is significantly higher in OSCC CSCs than in non-CSCs and this elevated expression is further correlated with advanced tumor stage and poor prognosis of OSCC patients, highlighting its clinical relevance in CSC-driven tumorigenesis (47, 48). Silencing lncRNA PVT1 markedly reduced the expression of CSC markers (CD24, BMI1, SOX2, ALDH1, and OCT4), and impaired CSC properties, as evidenced by diminished sphere-forming ability and tumorigenicity. Notably, the loss of PVT1 also attenuated the capacity of CSCs to initiate metastatic lesions *in vivo*, demonstrating that PVT1 sustains the dual attributes of stemness and metastatic potential​ in OSCC CSCs. Mechanistically, lncRNA PVT1 acts as a molecular sponge for miR-375, which relieves the post-transcriptional repression of its target gene yes-associated protein 1(YAP1), a key oncogenic effector in the Hippo signaling pathway. The subsequent upregulation of YAP1 promotes the expression of CSC-related genes, ultimately sustaining CSC stemness. Moreover, PVT1-mediated activation of the Hippo/YAP1 axis not only stabilizes CSC identity but also enhances the resistance of CSCs to chemotherapy, as YAP1 upregulates drug-efflux transporters and anti-apoptotic factors in CSCs (43).​ Furthermore, lncRNA PVT1 interacts with ​cellular myelocytomatosis oncogene (c-Myc) to potentiate its transcriptional activity, which in turn promotes CSC self-renewal and proliferation (20). This PVT1-c-Myc partnership also drives the metabolic reprogramming of CSCs, such as promoting glycolysis and glutamine metabolism, to fuel their sustained self - renewal and tumor - initiating capacity (49).

## 3 Clinical research on lncRNA PVT1 in OSCC patients

Beyond the well-documented functional roles of lncRNA PVT1 in OSCC pathogenesis from *in vitro* and *in vivo* studies, accumulating clinical evidence has further substantiated its significant clinical relevance. The following sections synthesize findings from human patient cohorts, focusing on the associations between lncRNA PVT1 expression and key clinicopathological parameters, patient survival outcomes, and treatment resistance, thereby bridging experimental insights with direct clinical implications.

### 3.1 Association of lncRNA PVT1 with clinicopathological features in OSCC

Multiple clinical studies have demonstrated a strong association between lncRNA PVT1 expression levels and the clinicopathological characteristics of patients with OSCC. For instance, in a study enrolling 68 OSCC patients, Wang and Zhang (2021) (38) reported that PVT1 expression was markedly higher in tumor tissues compared to adjacent normal tissues. Elevated PVT1 expression showed a positive correlation with​ advanced TNM stage (T3-T4 vs. T1-T2), higher pathological grade (Grade II+III vs. Grade I), and the presence of lymph node metastasis. Furthermore, preoperative plasma levels of PVT1 in OSCC patients were notably​ elevated relative to healthy controls and decreased markedly after surgery, suggesting its potential as a non-invasive biomarker for diagnosis and therapeutic monitoring (38). Another study comprising 83 OSCC patients revealed that PVT1 expression was substantially​ upregulated in tumor tissues compared with adjacent normal tissues. Moreover, elevated PVT1 expression was strongly associated with higher T classification (*P* = 0.041), advanced clinical stage (*P* < 0.001), and lymph node metastasis (*P* < 0.001) (17). In a separate cohort of 70 OSCC clinical samples, PVT1 levels were found to be markedly higher in metastatic OSCC tissues than in non-metastatic tissues, as well as in patients with stage III–IV disease relative to those with stage I–II disease (22). These findings collectively confirm that PVT1 expression levels are strongly indicative of the malignancy and progression status of OSCC.

### 3.2 ​Impact of lncRNA PVT1 expression on the survival outcomes of OSCC patients

The expression level of lncRNA PVT1 is closely associated with the prognosis of OSCC patients and may serve as a potential indicator for prognostic assessment. In a study of 83 OSCC patients, Yu et al. (2018) (19) demonstrated via Kaplan-Meier analysis that patients with high lncRNA PVT1 expression had markedly shorter overall survival (*P* < 0.05) as well as substantially shorter progression-free survival compared to those with low lncRNA PVT1 expression. Multivariate Cox regression analysis further confirmed that lncRNA PVT1 expression level is an independent prognostic factor for overall survival in OSCC patients (95% CI: 0.056–0.570, *P* = 0.004). Another study involving 44 OSCC patients also found that high lncRNA PVT1 expression was associated with notably worse overall survival (*P* = 0.003) and positively correlated with an increased risk of tumor recurrence (18). Additionally, follow-up data from 70 OSCC patients indicated that both recurrence-free survival and overall survival rates were significantly lower in the high lncRNA PVT1 expression group compared to the low expression group (*P* < 0.05) (22). These consistent results underscore the potential of lncRNA PVT1 as a valuable biomarker for prognostic evaluation, with implications for risk stratification in OSCC.

### 3.3 Role of lncRNA PVT1 in chemotherapy resistance of OSCC

The expression level of lncRNA PVT1 is closely associated with chemotherapy resistance in OSCC patients and may serve as a potential biomarker for predicting chemosensitivity. For instance, Wang et al. (2020) (17) divided 83 OSCC patients into cisplatin-resistant and cisplatin-sensitive groups. They observed that lncRNA PVT1 expression was considerably​ higher in tumor tissues of the cisplatin-resistant group compared to the sensitive group (*P* < 0.05). Furthermore, lncRNA PVT1 expression showed a negative correlation with miR-194-5p levels (*P* = 0.004) and a positive correlation with HIF1α expression (r = 0.6126, *P* < 0.0001). Additionally, Yang et al. (2021a) (45) reported that high lncRNA PVT1 expression in HNSCC tissues was strongly associated with resistance to cetuximab. Patients with elevated lncRNA PVT1 expression had significantly shorter overall survival after cetuximab treatment, suggesting that lncRNA PVT1 could also serve as a potential predictor of cetuximab treatment sensitivity in OSCC patients.

## 4 Clinical implications of lncRNA PVT1 in OSCC

As established in previous sections, the consistent upregulation of lncRNA PVT1 in OSCC tissues and patient plasma, along with its strong association with aggressive clinicopathological features (e.g., advanced TNM stage, lymph node metastasis) and poor survival outcomes, firmly supports its dual utility as a promising diagnostic and prognostic biomarker (17–38). Beyond its role as a biomarker, lncRNA PVT1 is a central driver of OSCC malignancy, making it a compelling therapeutic target. Current research on PVT1-targeted strategies is evolving and primarily explores several modalities (1): Nucleotide-based therapies, such as antisense oligonucleotides (ASOs) and small interfering RNAs (siRNAs), which directly degrade or inhibit PVT1 transcripts, have been shown to suppress OSCC cell proliferation and tumor growth in preclinical models (38–51) (2). Gene editing approaches​ like CRISPR/Cas9, which can knockout the lncRNA PVT1 locus to durably ablate its oncogenic function (52). (3) Small-molecule inhibitors​ that target key signaling pathways activated by lncRNA PVT1, such as the Wnt/β-catenin inhibitor iCRT14, which can reverse PVT1-mediated proliferation and EMT (19). In summary, lncRNA PVT1 represents a critical nexus in OSCC pathogenesis, bridging biomarker potential with therapeutic vulnerability. Future efforts should focus on validating these targeting strategies in advanced preclinical models and translating them into clinical trials, ultimately aiming to improve precision management for OSCC patients.

## 5 Discussion and prospects

OSCC, ranking as the sixth most common malignancy globally, is an aggressive cancer characterized by chemotherapy resistance, local invasion, and early lymph node metastasis (53–55), which leads to a five-year survival rate of less than 50% (53). Therefore, in-depth investigation into the therapeutic mechanisms and molecular targets of OSCC represents a current research priority.​ Exosomes, bilayered membrane vesicles (30–200 nm), are crucial for intercellular communication and homeostasis (56, 57). They transfer diverse RNA cargoes such as miRNAs, circular RNAs (circRNAs), lncRNAs and mRNAs from donor cells to OSCC cells, thereby modulating multiple malignant phenotypes, including proliferation, apoptosis, immune evasion, migration, and invasion (58). In a systematic review, Abdul et al. (2025) (59) identified several miRNAs, lncRNAs, and proteins as prognostic biomarkers for OSCC. Their expression levels demonstrated significant correlations with patient outcomes during OSCC progression. Notably, specific miRNAs, including miR-126, miR-130a, miR-155, and miR-21, are consistently associated with pathways that promote tumor growth, modulate the immune system, and regulate angiogenesis (59). CirRNAs exhibit stage-specific expression patterns in OSCC, showing significant correlations with clinical characteristics such as tumor size, distant metastasis, and TNM staging (60). Circular RNA PVT1 (circPVT1) is aberrantly expressed in OSCC and has recently been identified as a key regulator in this malignancy. Wang et al. (2022) (61) demonstrated that silencing circPVT1 suppresses the proliferation, migration, and invasion of OSCC cells by regulating miR-143-3p. Zhu (2020) (62) revealed that knockdown of circPVT1 suppresses the growth, metastasis, and glycolysis of OSCC cells by sequestering miR-106a-5p. These findings indicate that circPVT1 promotes the proliferation, migration, and invasion of OSCC cells. LncRNAs are differentially expressed in OSCC tissues and bodily fluids, where they participate in critical biological processes including cell proliferation, metastasis, metabolism, and drug resistance (63). Specific lncRNAs such as CHASERR, PVT1, and HASTER fine-tune the activity of dosage-sensitive genes encoding transcription factors, while others like lincRNA-p21 and Maenli contribute to the local activation of gene transcription (64). This review specifically focuses on how lncRNA PVT1 exerts its influence on OSCC by sponging microRNAs, including miR-185-5p, miR-7-5p, miR-150-5p, miR-194-5p, miR-124-3p, and miR-375. Collectively, these studies demonstrated that exosomes significantly impact OSCC progression, and intricate promoting or suppressive interactions exist among these regulatory molecules. In the pathogenesis of OSCC, lncRNA PVT1 drives tumor progression through multiple mechanisms, including promoting tumor cell proliferation, inhibiting apoptosis, enhancing invasive and metastatic capabilities, remodeling the tumor microenvironment, and inducing chemotherapy resistance. However, current research on the regulatory role of lncRNA PVT1 within the OSCC TME remains relatively limited, with existing findings primarily focused on its effects on cancer-associated fibroblasts and immune cell functions. Future studies should further investigate PVT1’s regulatory mechanisms concerning extracellular matrix remodeling and cytokine/chemokine networks. Notably, as the focus of oncology research evolves, the relationship between the gut microbiota and OSCC pathogenesis has emerged as a cutting-edge field, warranting exploration of PVT1’s potential involvement within this interdisciplinary context. Research has demonstrated an association between tumors and disruptions in gut microbiota composition, along with associated metabolic imbalances (65). Particularly intriguing is the intricate interplay among the gut microbiota, the TME, and immunotherapy efficacy. This interplay involves the gut microbiota modulating the TME, which in turn influences treatment effectiveness (66–68). However, whether lncRNA PVT1 participates in OSCC progression by influencing the gut microbiota-tumor microenvironment axis remains unexplored and represents a mechanism worthy of in-depth investigation.

Beyond the pivotal role of lncRNA PVT1, OSCC pathogenesis is orchestrated by a complex network of diverse non-coding RNAs (ncRNAs), each contributing uniquely to tumor initiation, progression, metastasis, and therapeutic resistance (69–71). Our understanding of this network has been substantially​ advanced by studies exploring salivary and tumor tissue ncRNAomes. For instance, other lncRNAs such as HOTAIR、H19 and MALAT1​ are frequently upregulated in OSCC saliva and tissues, with HOTAIR expression correlating strongly with lymph node metastasis, underscoring their potential as non-invasive prognostic biomarkers (72, 73). Similarly, miRNAs​ like miR-31、miR-27b and miR-21​ are not only implicated in OSCC proliferation and invasion but are also actively involved in mediating resistance to chemotherapeutic agents like cisplatin and 5-fluorouracil (74). Emerging players such as circRNAs add another layer of regulation. Salivary hsa_circ_0001874​ and hsa_circ_0001971​ are significantly elevated in OSCC patients and show correlation with tumor stage and grade, functioning as competitive endogenous RNAs (ceRNAs) to sponge miRNAs and activate oncogenic pathways (e.g., PLK1, SHP2) (75, 76). This ncRNA-mediated ceRNA network forms a dense regulatory circuit that fine-tunes gene expression critical for maintaining cancer stem cell properties, EMT, and metabolic reprogramming. Furthermore, the interplay between oral microbiota and host ncRNAs introduces an additional dimension to OSCC complexity. Pathogens like *Fusobacterium nucleatum* and *Porphyromonas gingivalis*can modulate the expression of specific lncRNAs (e.g., LINC00460, MIR4435-2HG, MX1-AS1) and bacterial small RNAs (e.g., sRNA23392), which in turn influence host cell invasion, immune evasion, and chemoresistance pathways (77–79). This highlights a pathogenic synergy where microbial dysbiosis and aberrant ncRNA expression converge to drive OSCC malignancy. In this intricate landscape, lncRNA PVT1 functions not in isolation but as a central hub within a broader ncRNA interactome. It exemplifies the common mechanisms—such as miRNA sponging (e.g., via the PVT1/miR-375/YAP1 axis) and transcription factor co-activation (e.g., with c-Myc)—that are employed by multiple ncRNAs to sustain oncogenic signaling. Therefore, while PVT1 is a critical regulator and a promising biomarker/therapeutic target, its full pathogenic significance is best understood within the context of the collaborative and often redundant ncRNA network that governs OSCC.

Currently, although significant progress has been made in understanding the functional mechanisms and clinical relevance of lncRNA PVT1 in OSCC, several challenges remain to be addressed. First, the precise molecular mechanisms by which lncRNA PVT1 regulates malignant behaviors in OSCC require further investigation. Key unanswered questions include the impact of m6A modifications on lncRNA PVT1 function and the interactive networks between lncRNA PVT1 and other lncRNAs or proteins. Second, existing clinical studies on lncRNA PVT1 are predominantly based on small sample sizes, underscoring the need for large-scale, multi-center clinical trials to validate the reliability of lncRNA PVT1 as a biomarker for diagnosis, prognosis assessment, and chemotherapy sensitivity prediction in OSCC. Furthermore, PVT1-targeted therapeutic strategies are still in the preclinical stage. Future efforts should focus on optimizing treatment regimens to enhance their safety and efficacy, thereby facilitating their transition to clinical applications.

Conclusively, lncRNA PVT1 is frequently upregulated in OSCC, where it orchestrates multiple malignant phenotypes, including proliferation, invasion, metastasis, immune evasion, chemoresistance, and cancer stem cell maintenance. LncRNA PVT1 expression levels are closely associated with clinicopathological features, patient prognosis, and chemotherapy sensitivity, underscoring its potential as a diagnostic, prognostic, and predictive biomarker. Moreover, therapies targeting lncRNA PVT1 represent a promising direction for precision medicine in OSCC, with significant translational potential. Moving forward, efforts should focus on elucidating the detailed mechanisms of lncRNA PVT1 in OSCC, validating its clinical utility in large, multi-center cohorts, and advancing its applications in precision oncology. Future therapeutic strategies may benefit from targeting such key nodal points (like PVT1) or, more broadly, disrupting the dynamic interactions within the entire ncRNA regulatory web.

## 6 Contribution and limitations

In concluding this review, a balanced consideration of its contributions and limitations is warranted. While offering a systematic synthesis, this work is subject to inherent constraints. Its conclusions are derived from existing literature, meaning they depend on the methodological quality and scope of the cited studies, and lack direct experimental validation. The clinical correlations presented are predominantly based on retrospective studies with limited sample sizes, and proposed connections to emerging fields, such as the gut microbiome, require further empirical substantiation. These limitations highlight critical avenues for future research, including large-scale prospective studies and deeper mechanistic exploration.

Nevertheless, this review makes distinct and valuable contributions. First, it provides a holistic synthesis. It moves beyond fragmented discussions to present a comprehensive portrait of the multifaceted oncogenic roles of PVT1 in OSCC, integrating its functions in proliferation, apoptosis, metastasis, immune evasion, chemoresistance, and stemness maintenance. Second, it adopts a network-oriented perspective. It contextualizes PVT1 not as an isolated molecule but as a central hub within the complex regulatory network of ncRNAs, offering a systems-level understanding of OSCC pathogenesis. Third, it is forward-looking and translation-focused. It not only consolidates current knowledge but also explicitly identifies translational bottlenecks and potential strategies—from refining targeted therapies to exploring interdisciplinary mechanisms, thereby outlining a feasible pathway for leveraging PVT1 in the precision management of OSCC.
